# Identification of Diagnostic Biomarkers for Myocardial Infarction Using Bioinformatics and Disulfidptosis-Targeted Computational Drug Discovery

**DOI:** 10.1155/mi/5054377

**Published:** 2025-09-02

**Authors:** Haoran Zhang, Ziguang Song, Weitao Shen, Donghui Zhang

**Affiliations:** ^1^Cardiovascular Medicine Ward, The Second Affiliated Hospital of Harbin Medical University, Harbin, China; ^2^Cardiovascular Medicine Ward, The First Affiliated Hospital of Harbin Medical University, Harbin, China; ^3^Bioinformatics R&D Department, Hangzhou Mugu Technology Co., Ltd., Hangzhou, China

**Keywords:** biomarker, disulfidptosis, immune regulation, machine learning, myocardial infarction, predictive drug

## Abstract

Disulfidptosis, a newly discovered form of regulated cell death, is involved in multiple disease processes. This study applied computational methods to identify disulfidptosis-related genes in myocardial infarction (MI). Differentially expressed genes (DEGs) from GSE66360 dataset were screened using the limma package and intersected with genes in weighted gene coexpression network analysis (WGCNA) modules to obtain candidate genes. Biomarkers were selected via support vector machine-recursive feature elimination (SVM-RFE) and least absolute shrinkage and selection operator (LASSO), and validated by quantitative real-time (qRT)-PCR, CCK-8, and flow cytometry. Enrichment and immune infiltration analyses were performed using clusterProfiler and CIBERSORT tools. Potential drugs were predicted via the Coremine database and visualized with Cytoscape. Seurat and CellChat packages were employed to perform single-cell transcriptomic analysis and develop cell–cell communication network, respectively. The genes in the lightgreen module that had the highest correlation with immune scores were selected. Next, we identified 10 biomarkers (*THBD*, *IRAK3*, *NFIL3*, *IL1R2*, *THBS1*, *MAP3K8*, *JDP2*, *FCGR2A*, *CCL20*, and *EREG*), all of which showed significantly higher mRNA levels in AC16-oxygen–glucose deprivation (OGD) cells compared to controls. Silencing *MAP3K8* and *NFIL3* enhanced cell viability and reduced apoptosis in AC16-OGD cells. Immune infiltration analysis suggested that *NFIL3* and *MAP3K8* modulated T cell function, contributing to MI pathogenesis. Drug analysis predicted 15 candidate drugs targeting both *NFIL3* and *MAP3K8*. Single-cell analysis showed that distinguished six cell types in MI, with adipocytes serving as a communication hub interacting closely with cardiomyocytes, fibroblasts, endothelial cells, and macrophages. These findings highlighted the potential of the identified biomarkers as novel therapeutic targets for MI.

## 1. Introduction

Myocardial infarction (MI) is an acute myocardial ischemia and hypoxia condition caused by coronary atherosclerosis, representing the most severe coronary artery disease (CAD) [1–3]. Statistics showed that among 19,781 CAD patients, MI affects 23.3% of CAD patients [4], with a 20% recurrence rate within the first incidence of MI [5]. Despite therapeutic advancements, MI remains a leading global cause of morbidity and mortality [6]. Cardiac troponin (cTn), which has high sensitivity and specificity, is currently the standard method for diagnosing MI [7]. Though circulating cTn levels can directly reflect myocardial injury severity [8], research indicated that cTn levels are elevated in individuals with heart failure (HF), sepsis, and chronic kidney disease, showing the potential to increase risk of false-positive diagnosis [9]. The complex pathophysiology of MI points to the need for more reliable biomarkers. Identifying novel molecular gene signature could deepen our understanding of MI pathogenesis and contribute to targeted therapies [10, 11].

Disulfidptosis involves sulfide-induced stress response within cells [12, 13]. This process plays a critical role in maintaining cellular redox balance, particularly in a variety of tumors [14]. Glucose deprivation in cells with a high protein expression of *SLC7A11* causes NADPH depletion [13, 15], leading to the formation of disulfide bonds in actin cytoskeleton and subsequent disulfide-induced apoptosis [16]. Previous bioinformatics analysis identified disulfidptosis-related markers in AIM, and found that INF2 and CD2AP have potential prognostic value [17]. However, the underlying mechanisms of the role of disulfidptosis in MI still remain to be elucidated, especially its effects on immune cell infiltration and MI [18].

Here, we aimed to screen early diagnostic biomarkers associated with disulfidptosis for MI and to explore their potential mechanisms in the immune microenvironment. In vitro experiments were also carried out to validate the identified biomarkers. Furthermore, the regulatory roles of disulfidptosis-correlated genes and immune infiltration in the onset and progression of MI were analyzed and potential therapeutic targets were predicted, providing novel insights for the prevention of MI and its treatment. Importantly, this study was the first to systematically integrate bulk and single-cell transcriptomic data to investigate the cell-type-specific expression patterns and intercellular communication of disulfidptosis-related genes in MI. These findings not only enriched our understanding of disulfidptosis in cardiovascular disease, but also offer a theoretical foundation and candidate targets for precise diagnosis and immunomodulatory therapy of MI.

## 2. Materials and Methods

### 2.1. Acquisition of Data

This study was performed based on the data from the GSE66360 dataset (50 control samples and 49 MI samples) in the Gene Expression Omnibus (GEO, http://www.ncbi.nlm.nih.gov/geo/) database on the GPL570 platform. The information on a total of 1793 immune-related genes (IRGs) was collected from the ImmPort database (https://www.immport.org/shared/). Additionally, this study obtained 12 disulfidptosis-related genes (*NCKAP1*, *WASF2*, *RAC1*, *NUBPL*, *SLC7A11*, *SLC3A2*, *RPN1*, *NDUFS1*, *NDUFA11*, *GYS1*, *LRPPRC*, and *OXSM*) from a published literature [13].

### 2.2. Identification of Differentially Expressed Genes (DEGs)

The limma package [19, 20] was employed to identify DEGs based on the gene expression data of MI and control samples in the GSE66360 dataset under the screening criteria of *p* < 0.05 and |log_2_FC| > 1.

### 2.3. Weighted Gene Coexpression Network Analysis (WGCNA)

First, the ImmuneScore of samples was calculated by the single-sample GSEA (ssGSEA) algorithm [21]. As a systematic biological method, WGCNA reveals the correlation patterns among the genes across different samples by classifying coexpression modules with scale-free network characteristics. To ensure a scale-free nature of the network, the pickSoftThreshold function was employed to determine appropriate soft threshold β. Subsequently, the expression matrix was transformed into an adjacency matrix, which was then converted into a topological overlap matrix (TOM). Next, genes were clustered by average-linkage hierarchical clustering method based on TOM. After determining gene modules through dynamic tree cutting, the eigengene values for each module were calculated. Next, cluster analysis was conducted to merge similar modules into new ones under the settings of height = 0.25, deepSplit = 2, and minModuleSize = 50.

### 2.4. Machine Learning for Biomarker Selection

The module genes with the highest significance were included in subsequent analysis. Utilizing the recursive feature elimination (RFE) function in the Caret *R* package [22] and the svmLinear method of support vector machine (SVM), the number of features was selected by RFE. Subsequently, the *R* package glmnet was used to screen MI-associated markers with 10-fold crossvalidation (CV). Feature factors were identified by logistic regression with key parameters of *n*folds = 10 and family = “binomial”. Feature genes selected by both SVM and least absolute shrinkage and selection operator (LASSO) methods were intersected to obtain common genes as MI-correlated biomarkers. Finally, the predictive power of these biomarkers was evaluated using receiver operating characteristic (ROC) curves.

### 2.5. Cell Incubation and Transfection

To validate the obtained biomarkers, we conducted experimental validation using human cardiomyocytes AC16 purchased from the BeNa Culture Collection (BNCC339980, Henan, China). The cells were cultured in DMEM/F12 growth medium supplemented with HEPES (C3270, Solarbio Lifesciences, Beijing, China), 1% penicillin/streptomycin (P/S, Gibco, Waltham, MA), and 10% fetal bovine serum (FBS, Thermo Fisher Scientific, Waltham, MA). Constant temperature at 37°C and 5% CO_2_ concentration were maintained to ensure the optimal growth conditions for all the cells. STR identification was carried out to authenticate the cell line, and the outcome of mycoplasma detection was negativity.

The small intern RNA (siRNA) was synthesized by GenePharma (Shanghai, China) and transfected into cells utilizing Lipofectamine 2000 (11668027, Invitrogen, Carlsbad, CA) according to the instructions. The siRNA sequences used were as follows: 5′-AGCACTTTATGAGCTTGAACTCT-3′ (si-*MAP3K8*) and 5′- CCCAACTTCATTCAATAAGGAGC-3′ (si-*NFIL3*).

### 2.6. Oxygen–Glucose Deprivation (OGD) Cell Models

After the cells reached the logarithmic growth phase, the culture flask was removed from the incubator and gently tilted to aspirate the medium using a pipette. To create an OGD cell model [23], the AC16 cells were washed with prewarmed (37°C) phosphate-buffered saline (PBS, ThermoFisher Scientific) and added with an appropriate volume of sugar-free DMEM/F12 medium containing HEPES, 10% FBS, and 1% P/S, ensuring complete coverage of the cells. Subsequently, the cells were placed into a hypoxia chamber (Billups–Rothenberg, San Diego, CA, USA), which was flushed with gas mixture of 94% N_2_, 5% CO_2_ and 1% O_2_ for 30 min. After sealing the chamber, the cells were further incubated for 24 h. During the cell culture, the culture plates were regularly observed under an Eclipse Ts2R microscope (Nikon, Tokyo, Japan) to assess the cell morphology and confirm successful establishment of AC16-OGD cell model. The AC16-control group was cultured in regular medium under normal oxygen condition.

### 2.7. Quantitative Real-Time PCR (qRT-PCR)

Total RNA was isolated from cellular samples using TRIzol reagent (Invitrogen, Carlsbad, CA) and then efficiently converted into cDNA employing the PrimeScript RT reagent Kit with a *g*DNA Eraser (Takara, Kyoto, Japan) [24]. The cDNA served as the template for subsequent qRT-PCR experiment. Using the fast Real-Time PCR 7500 system (Applied Biosystems, Foster City, CA), the expression profiles of 10 biomarkers associated with the disulfidptosis score (*THBD*, *IRAK3*, *NFIL3*, *IL1R2*, *THBS1*, *MAP3K8*, *JDP2*, *FCGR2A*, *CCL20*, and *EREG*) in the control and OGD cell groups were measured. The PCR amplification began with an initial denaturation step at 50°C for 2 min, followed by 40 cycles with each cycle consisting denaturation at 95°C for 15 s and annealing at 60°C for 1 min. *GAPDH*was an internal control gene for normalization. The expression of each biomarker was calculated using the 2^−ΔΔCt^ method and visually presented by bar graph. The qRT-PCR primers were designed based on the NCBI sequences in Primer Premier 6 software (Supporting Information 1: Table S1). The GAPDH reference gene sequence was obtained from OriGene (https://www.origene.com.cn/).

### 2.8. Cell Viability Assay

Transfected AC16-OGD cells at the concentration of 2 × 10^3^ cells/well were cultured in a 96-well plate for 48 h before 4-h treatment with 10 μL CCK-8 solution (C0037, Beyotime, China). The viability of transfected AC16-OGD cells was calculated by reading the optical density at 450 nm with an iMark microplate reader (Bio-Rad, Hercules, CA).

### 2.9. Flow Cytometry

According to the instructions, 195 μL of annexin-V FITC (BD Biosciences, Franklin Lakes, NJ) with 5 μL of propidium iodide was used to resuspend the PBS-rinsed AC16-OGD cells. Next, following incubation at ambient temperature in the dark for 10 min, flow cytometry was performed. Lysis software (EPICS-XL, Ramsey, Minnesota, USA) was employed for data analysis.

### 2.10. ssGSEA

The gene expression matrix in the GSE66360 dataset was selected and the expression data of 12 disulfidptosis-related genes were extracted. The ssGSEA method with the GSVA *R* package was employed to calculate the disulfidptosis score for each sample. Prior to the ssGSEA calculation, quality control was performed by filtering out low-expression genes, that showed minimal expression across most samples, thereby reducing background noise. The method assigns sample-specific enrichment scores that reflect the overall activity of the biological process in each sample. Next, Pearson correlation analysis was used to assess the relationship between disulfidptosis scores and hub genes in MI samples.

### 2.11. Immune Infiltration Analysis and Gene Enrichment Analysis

The CIBERSORT method [25, 26] was applied to assess the immune infiltration in the MI and control groups in GSE66360 dataset. Furthermore, to uncover the functional roles of the identified DEGs and modular genes, the clusterProfiler *R* package [27] was utilized to conduct Gene Ontology (GO)-biological process (BP) and Kyoto Encyclopedia of Genes and Genomes (KEGG) enrichment analyses.

### 2.12. Construction of Drug–Gene Interaction Network

Based on the Coremine database (https://coremine.com/medical), drugs that can regulate the target gene were predicted, and those with a *p* < 0.01 were selected to develop a target-drug network using Cytoscape software.

### 2.13. Single-Cell RNA Sequencing (scRNA-Seq) Analysis for MI

The scRNA sequencing dataset GSE270788, which contained heart tissue samples from five acute myocardial infarction (AMI) patients and seven healthy donors, was collected from the GEO database (https://www.ncbi.nlm.nih.gov/geo/). Quality control was performed using the Seurat package [28] to retain cells with mitochondrial gene content < 7.5% and 200–3000 detected genes, resulting in a total of 28,619 cells. The data were normalized with the ScaleData function, followed by principal component analysis (PCA) for dimensionality reduction. Batch effects across the samples were corrected using the Harmony package. UMAP visualization and clustering (resolution = 0.2) were conducted based on the top 20 PCs using the FindNeighbors and FindClusters functions. Cell types were annotated according to the expressions of the known marker genes from the CellMarker2.0 database [29]. In addition, high-expressed genes specific to each cell subpopulation in MI (only.pos = TRUE, min.pct = 0.25, logfc.threshold = 0.25) were calculated by the FindAllMarkers function and then subjected to pathway enrichment analysis using the compareCluster function of the clusterProfiler package. Finally, to explore the signaling characteristics of disulfidptosis-associated cells in MI, we used the CellChat package to conduct intercellular ligand-receptor interactions analysis [30].

### 2.14. Statistical Analysis

All statistical data were analyzed in *R* language (version 3.6.0). Wilcoxon test was employed to calculate the differences between two groups of continuous variables. A correlation matrix between the disulfidptosis score and biomarkers in MI samples was constructed using the Pearson method, with a *p* < 0.05 denoting statistical significance. GraphPad Prism software (version 8.0.2) was employed for analyzing the experimental data, which were compared using analysis of variance or unpaired t test. Sangerbox (http://sangerbox.com/) offered analytical assistance [31].

## 3. Results

### 3.1. The Selection of DEGs

**Figure 1:**
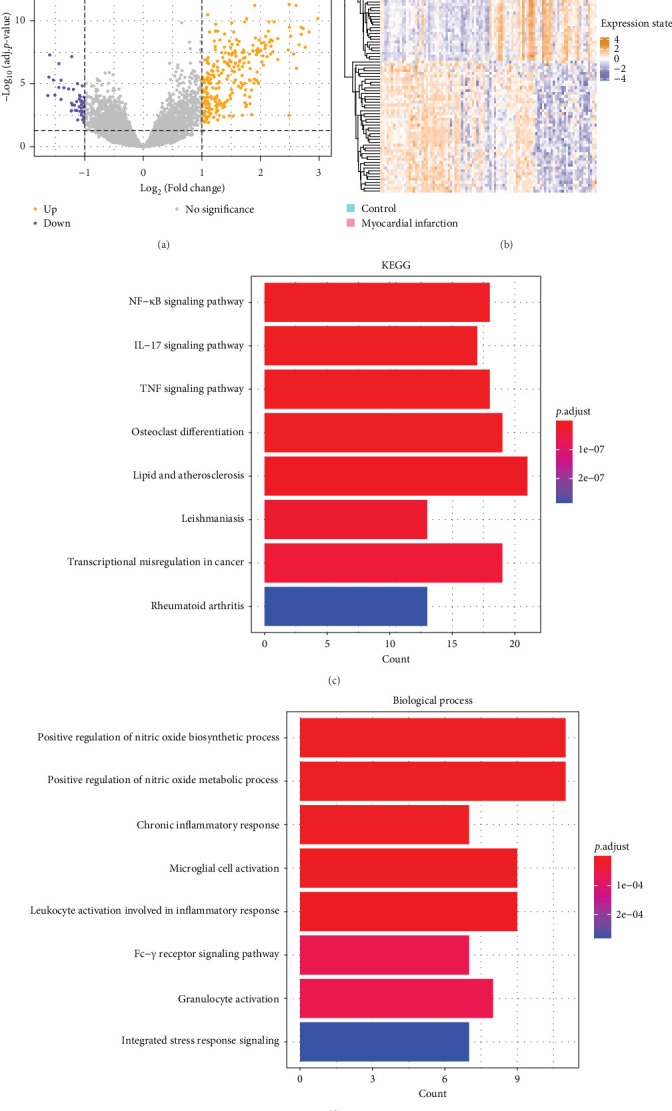
Screening and the enrichment analysis of DEGs. (A) Volcano plot of DEGs, with purple dots representing significantly downregulated genes and yellow dots representing significantly upregulated genes. (B) Heatmap of DEGs. (C) KEGG pathway enrichment analysis of DEGs. (D) GO–BP enrichment analysis of DEGs.

Differential gene expression analysis was conducted to obtain DEGs between MI samples and control samples (Figure 1A,B). KEGG pathway enrichment analysis on the upregulated DEGs showed that these genes were primarily enriched in pathways including IL−17 signaling pathway and nuclear factor (NF)-*κ*B signaling pathway (Figure 1C). Meanwhile, the GO analysis demonstrated that the DEGs were enriched in pathways including positive regulation of nitric oxide biosynthetic/metabolic process, chronic inflammatory response, and microglial cell activation (Figure 1D).

### 3.2. Identifying Gene Modules Correlated With Immune Regulation Through WGCNA Analysis and ImmuneScores

**Figure 2:**
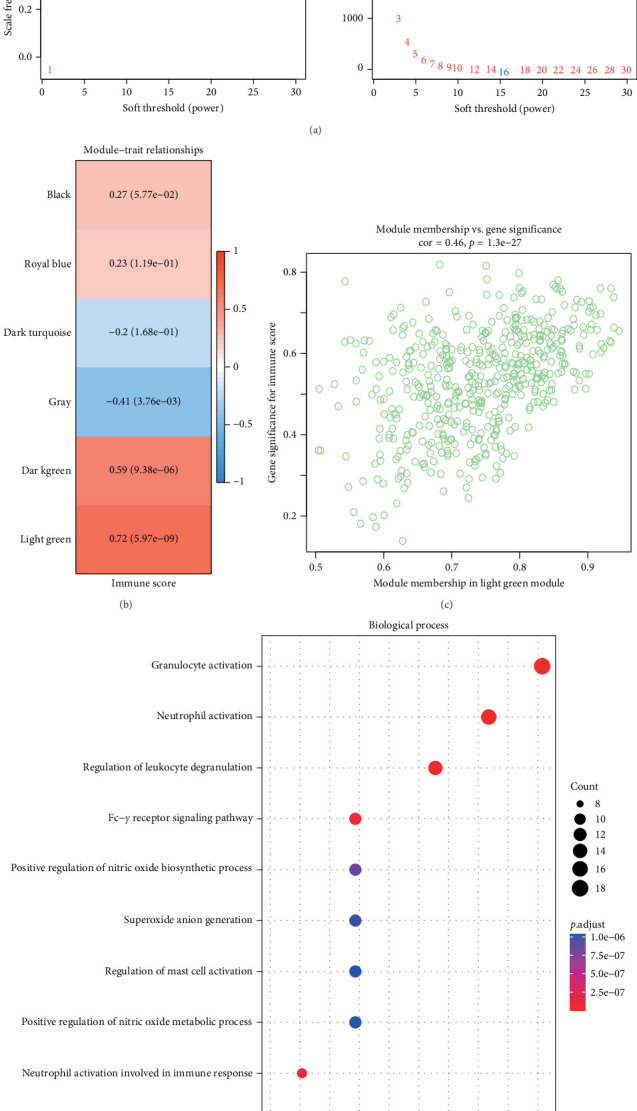
The results of WGCNA. (A) Analysis of network topology characteristics under different soft-thresholding powers. The left plot depicts the scale-free topology characteristic, while the right plot shows the average connectivity. (B) Heatmap of correlation between modules and ImmuneScores, with Pearson correlation coefficients and their significance levels between each color module and the ImmuneScores indicated in the corresponding cells. (C) Scatter plot representing gene module membership and gene significance for the lightgreen module. (D) GO–BP enrichment analysis results for genes in the light green module.

Gene modules correlated with immune regulation were identified based on the results of ssGSEA and WGCNA analysis.  Figure 2A shows the network topological properties under different soft-thresholding powers in WGCNA. The left panel demonstrated that the scale independence of the network gradually stabilized and approached 1 with increasing soft-thresholding power, indicating the establishment of a scale-free network topology. The right panel presented the trend of mean connectivity with increasing soft-thresholding power, indicating a gradual decrease in mean connectivity with higher soft-thresholding powers. A soft-threshold *β* = 16 was chosen to ensure a scale-free network. Next, hierarchical clustering identified six coexpression modules after merging, and the gray module contained genes that could not be clustered into other modules. The correlation heatmap between each gene module and the ImmuneScore showed that the lightgreen module exhibited the highest positive association with the ImmuneScore (cor = 0.72, *p*= 3.6e-9, Figure 2B). Moreover, a positive connection between gene significance and gene module membership within the lightgreen module (cor = 0.46, *p*= 1.3e-27) was observed, suggesting that the genes in this module were also closely related to the ImmuneScore (Figure 2C). GO enrichment analysis further revealed that the lightgreen module genes were enriched in BP terms including granulocyte activation, neutrophil activation, and regulation of leukocyte degranulation (Figure 2D).

### 3.3. Machine Learning Analysis for Screening MI Diagnostic Biomarkers

**Figure 3:**
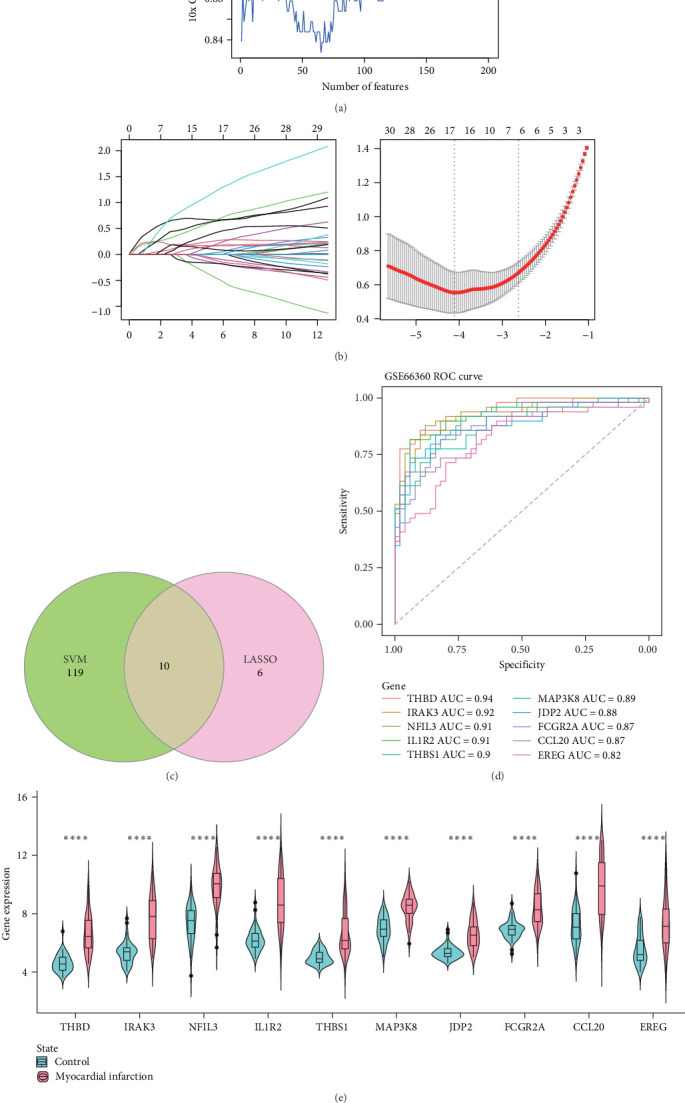
Screening of biomarkers using machine learning. (A) The curve of crossvalidation accuracy varying with the number of features selected by the RFE method in the SVM model. (B) The variation of regression coefficients for gene features in the LASSO regression model and the optimal penalty parameter (*λ*) determined through crossvalidation. (C) A Venn diagram displaying the intersection of feature genes selected by both SVM and LASSO methods, resulting in a total of 10 biomarkers. (D) The ROC curves and corresponding AUC values for the 10 biomarkers in the GSE66360 dataset. (E) Violin plots showing the expression distribution of the 10 biomarkers in the normal control group and the MI group, with *⁣*^*∗∗∗∗*^ indicating *p* < 0.0001.

Based on the genes from the lightgreen module, the CV accuracy trend of the SVM model showed the highest accuracy when there were 129 selected features (Figure 3A). Gene selection was further performed using LASSO regression analysis. The left panel of Figure 3B presented changes in the regression coefficients for different gene features in LASSO regression with varying penalty parameters (*λ*). The red dashed line in the right panel of Figure 3B represented the optimal *λ* value selected by 10-fold CV. The optimal *λ* value corresponded to a relatively small number of features, while maintaining strong model predictive performance.

A total of 10 genes, namely, *THBD*, *IRAK3*, *NFIL3*, *IL1R2*, *THBS1*, *MAP3K8*, *JDP2*, *FCGR2A*, *CCL20*, and *EREG*, were in the intersection of the feature genes selected by SVM (129 genes) and LASSO (16 genes) methods (Figure 3C) and were considered as the biomarkers in this study. The area under curve (AUC) values for these 10 biomarkers were all above 0.7, indicating their high predictive power in MI (Figure 3D). Further analysis of the expression differences of these 10 biomarkers between the MI group and control group revealed that these genes were all high-expressed in the MI group (*p* < 0.0001, Figure 3E).

### 3.4. Identification of Biomarkers Related to Disulfidptosis and Genes Involved in Immune Regulation

**Figure 4:**
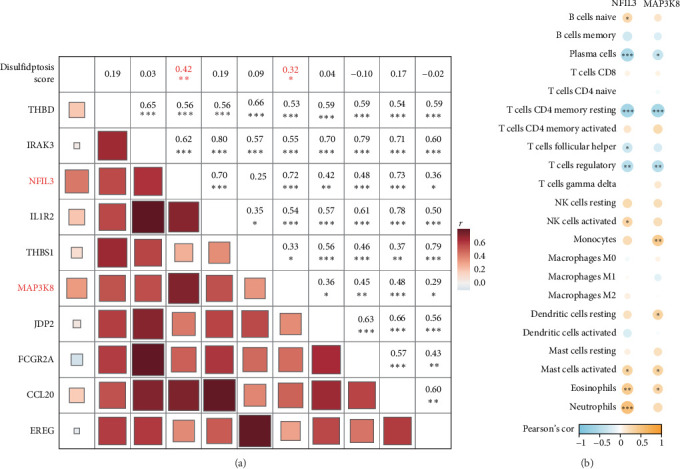
Identification of biomarkers associated with disulfidptosis and immune regulatory genes. (A) Pearson correlation matrix between disulfidptosis score and 10 biomarkers. (B) Pearson correlation analysis results between *NFIL3* and *MAP3K8* genes and different immune cell subsets. Each point represents the Pearson correlation coefficient between a gene and a specific immune cell subset, with the size of the point indicating the strength of the correlation. The color ranges from blue to orange, representing the correlation from negative to positive. *⁣*^*∗∗∗*^*p* < 0.001, *⁣*^*∗∗*^*p* < 0.01, and *⁣*^*∗*^*p* < 0.05.

Pearson correlation matrix was constructed to evaluate the relationships between the disulfidptosis score and the 10 biomarkers in MI samples. Most genes exhibited significant positive correlations (*p* < 0.05), with *NFIL3* (correlation coefficient of 0.42) and *MAP3K8* (correlation coefficient of 0.32) demonstrating particularly strong associations with the disulfidptosis score (*p* < 0.05, Figure 4A). The correlation analysis between *NFIL3*, *MAP3K8*, and different immune cell subsets showed that *NFIL3* was closely inversely correlated with multiple immune cell subsets, such as T cells CD4 memory resting, plasma cells, T cells regulatory, and T cells follicular helper (*p* < 0.05, Figure 4B) but significantly positively linked to natural killer (NK) cells activated, neutrophils, mast cells activated, B cells naïve, and eosinophils (*p* < 0.05, Figure 4B). Similarly, *MAP3K8* was also significantly negative correlated with T cells CD4 memory resting, plasma cells, T cells regulatory (*p* < 0.05) but strongly positively correlated with mast cells activated, monocytes, dendritic cells resting, and eosinophils (*p* < 0.05, Figure 4B).

### 3.5. In Vitro Validation of the Biomarkers

**Figure 5:**
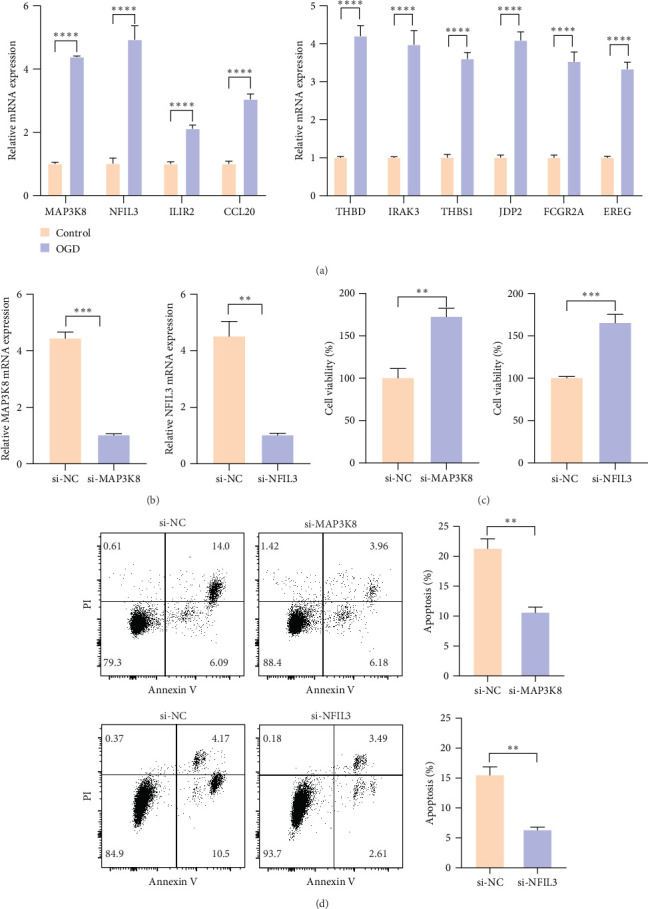
In vitro experimental validation of biomarkers. (A) Relative mRNA expression levels of 10 biomarkers (*MAP3K8*, *NFIL3*, *IL1R2*, *CCL20*, *THBD*, *IRAK3*, *THBS1*, *JDP2*, *FCGR2A*, and *EREG*) in human cardiomyocyte AC16-control and AC16-OGD cells, assessed via qRT-PCR. (B) qRT-PCR was used to validate the knockout efficiency of *MAP3K8* and *NFIL3*. (C) The percentage of cell viability in the si-*MAP3K8* and si-*NFIL3* group compared to the si-NC group. (D) Flow cytometry was used to examine the apoptosis of si-*MAP3K8* and si-*NFIL3* group compared to the si-NC group. All the data from three independent tests were shown as mean ± standard deviation (SD). *⁣*^*∗∗∗∗*^*p* < 0.0001; *⁣*^*∗∗∗*^*p* < 0.001; and *⁣*^*∗∗*^*p* < 0.01. Three independent repetitive tests were used in all procedures.

To further verify the expressions of the screened markers in MI, the qRT-PCR data showed that the mRNA expressions of all the 10 markers (*THBD*, *IRAK3*, *NFIL3*, *IL1R2*, *THBS1*, *MAP3K8*, *JDP2*, *FCGR2A*, *CCL20*, and *EREG*) were significantly upregulated in AC16-OGD cells than in control AC16 cells (Figure 5A, *p* < 0.001). Since *NFIL3* and *MAP3K8* were closely related to disulfidptosis score, these two genes were chosen for the validation of cellular functions *in vitro* (Figure 5B). We observed significantly enhanced viability of AC16-OGD cells after silencing these two genes (Figure 5C, *p* < 0.01). Additionally, flow cytometry showed that silencing *NFIL3* and *MAP3K8* markedly inhibited apoptosis in AC16-OGD cells (Figure 5D, *p* < 0.05).

### 3.6. Relationship Between Immune Infiltration and *NFIL3* and *MAP3K8* in MI

**Figure 6:**
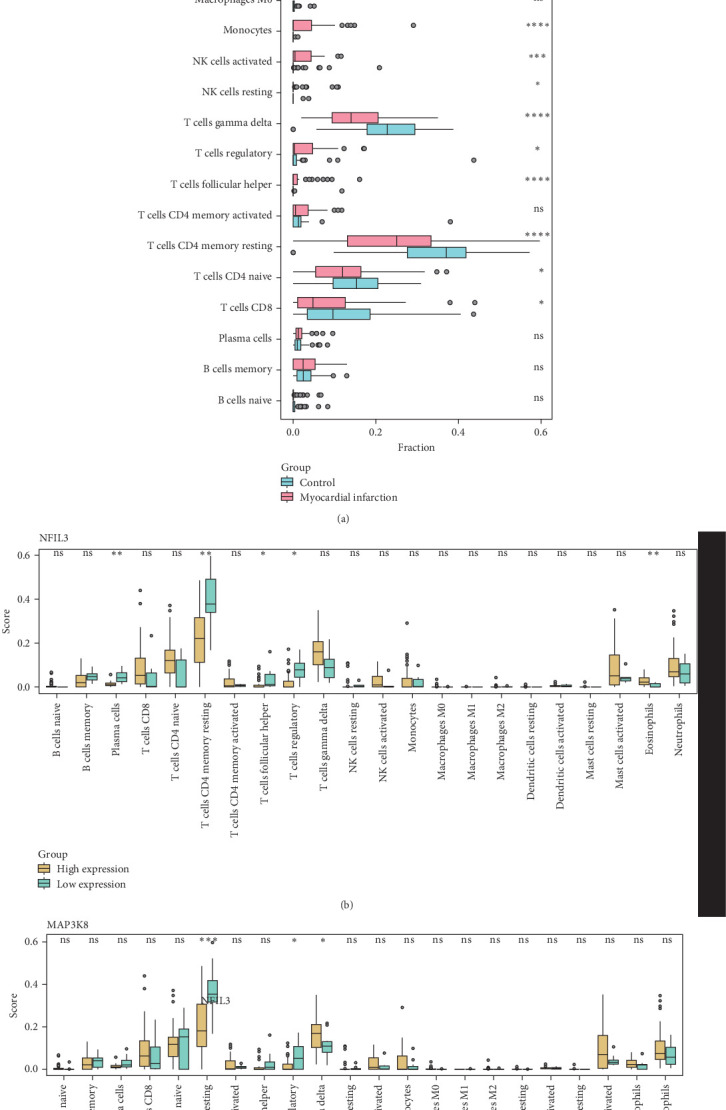
Relationship between immune infiltration and NFIL3 and MAP3K8 in MI. (A) Differences in the proportions of various immune cell subpopulations between the MI group and the control group in the GSE66360 dataset. (B) Differences in the proportions of immune cell subpopulations between the two groups with different *NFIL3* expressions. (C) Differences in the proportions of immune cell subpopulations between the two groups with different *MAP3K8* gene expression levels. *⁣*^*∗*^*p* < 0.05, *⁣*^*∗∗*^*p* < 0.01, *⁣*^*∗∗∗*^*p* < 0.001, and *⁣*^*∗∗∗∗*^*p* < 0.0001.

Analysis of the proportions of various immune cell subpopulations between the MI group and the control group in the GSE66360 dataset showed that the number of mast cells activated, neutrophils, NK cells activated, eosinophils, and monocytes was significantly increased in the MI group (Figure 6A, *p* < 0.05), while the proportions of T cells CD4 memory resting, T cells gamma delta, T cells CD8, T cells gamma delta, and T cells CD4 naive were significantly reduced (Figure 6A, *p* < 0.05). This indicated a possible correlation between MI and the dynamic changes of T cells. Further analysis showed that samples with high-expressed *NFIL3* had notably increased proportion of immune cells, such as eosinophils (Figure 6B, *p* < 0.05) but lower proportion of other types of cells, such as T cells regulatory, T cells follicular helper, T cells CD4 memory resting, plasma cells (Figure 6B, *p* < 0.05). In samples with high expression of *MAP3K8*, the proportion of T cells gamma delta was markedly higher (Figure 6C, *p* < 0.05), while that of other immune cells including T cells regulatory and T cells CD4 memory resting was reduced (Figure 6C, *p* < 0.05).

### 3.7. Prediction of Drugs That Can Regulated Target Genes Based on the Coremine Database

**Figure 7:**
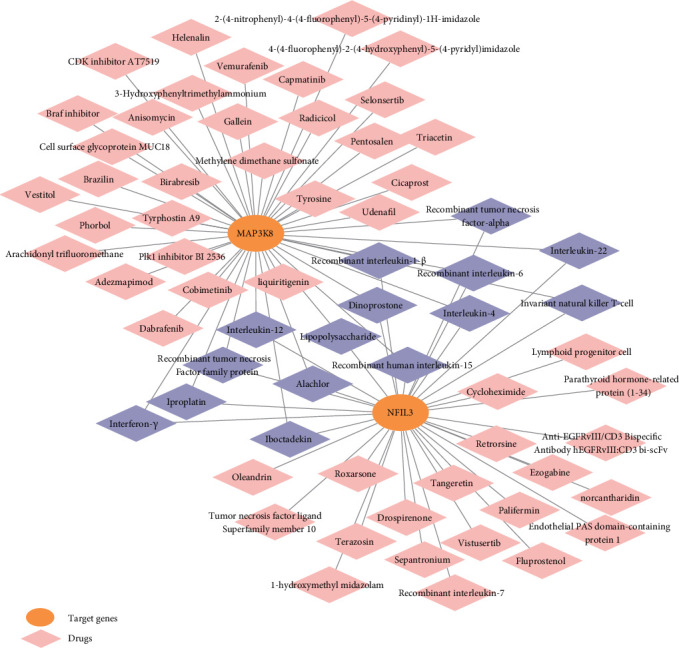
The relationships between *MAP3K8*, *NFIL3*, and various drugs or compounds associated with them. The orange circular nodes represent the target genes, while the diamond nodes represent the drugs related to these genes. The purple diamonds specifically highlight the drugs that are associated with both genes.

We first obtained drugs that can modulate the target genes from the Coremine database. Drugs with *p* < 0.01 were selected to develop a target-drug network using Cytoscape software. We found a total of 15 drugs significantly associated with both *NFIL3* and *MAP3K8* (Figure 7, *p* < 0.01).

### 3.8. The scRNA-Seq Analysis and Intercellular Communication in MI

**Figure 8:**
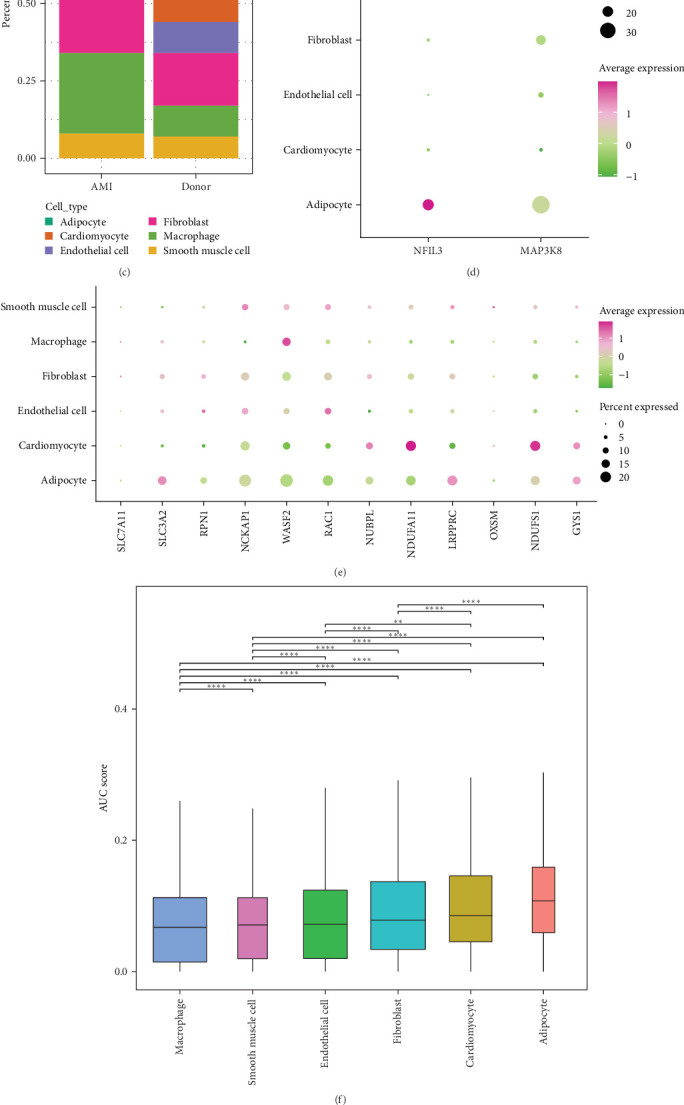
Single-cell mapping of MI based on the GSE270788 dataset. (A) A total of six cell types (including adipocytes, smooth muscle cells, endothelial cells, fibroblasts, cardiomyocytes, and macrophages) were identified based on scRNA-seq data from MI and its donor samples. (B) Bubble plots to demonstrate the expressions of the marker genes in the six cell types. (C) Differences in the proportion of different cell types occupied between MI and donor samples. (D) The expression levels of the key genes *NFIL3* and *MAP3K8* were demonstrated in six cell types, respectively. (E) Expression map of disulfidptosis-related genes in different cell types. (F) The AUC score reflects the degree to which each cell responds to the disulfidptosis signature. (G) Bioprocess enrichment analysis of disulfidptosis high versus low expression samples.

Using the GSE270788 dataset, we constructed a single-cell atlas of MI. After dimensionality reduction and clustering analysis of the scRNA-seq data, a total of six cell types were identified, including adipocytes, macrophages, cardiomyocytes, smooth muscle cells, endothelial cells, and fibroblasts (Figure 8A). The proportion of marker genes for different cell types and different cells is shown in Figure 8B,C. We found that *NFIL3* was predominantly expressed in adipocytes, while *MAP3K8* was predominantly expressed in macrophages (Figure 8D). Several disulfidptosis-related genes were also expressed in different MI cell types. Specifically, cardiomyocytes mainly expressed genes, such as *NDUFA11*, *NDUFS1*, and *GYS1*; macrophages mainly expressed *WASF2*; adipocytes mainly expressed genes, such as *SLC3A2* and *LRPPRC* (Figure 8E). As shown in Figure 8F, we used the AUCell package to calculate the activity scores for the disulfidptosis gene set in each cell, and observed that the adipocyte subpopulation had higher activity scores (*p* < 0.0001). Based on the median AUC score, the adipocyte subpopulation was divided into HIGH and LOW expression groups, and differential genes in the cells of the ineligible groups were calculated by the FindMarkers function. The enrichment results indicated that the groups with higher AUC scores were mainly enriched in biological processes (BPs), such as muscle cell development and heart contraction (Figure 8G).

**Figure 9:**
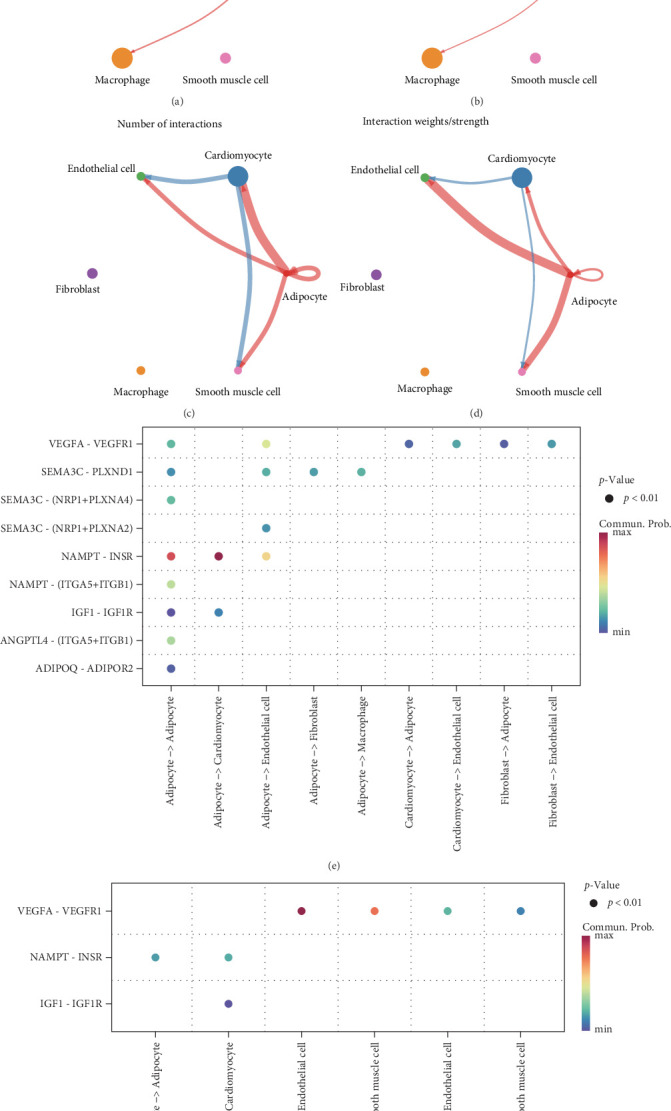
Analysis of intercellular communication between six cell subpopulations in MI. (A, B) Number of interactions (A) and interaction strength (B) between cells in MI samples. (C, D) Number of interactions (C) and interaction strength (D) between cells in donor samples. (E, F) Ligand-receptor bubble diagram between cells in MI (E) and donor (F) samples.

In addition, we conducted pathway enrichment analysis of high-expressed genes in the six cellular subpopulations using the compareCluster function of the clusterProfiler package. It was observed that the macrophage subpopulation was predominantly enriched in the binding and uptake of ligands by scavenger receptors pathways, while the adipocyte subpopulation was mainly associated with interleukin (IL) signaling, nuclear events, and NTRK signaling pathways (Supporting Information 2: Figure S1). Next, to characterize intercellular communication in disulfidptosis high-expressing cells during MI, we performed intercellular ligand-receptor interactions analysis based on the scRNA-seq data using the CellChat package. It was found that in MI samples, adipocytes acted as communication hubs and established frequent signaling connections with cardiomyocytes, fibroblasts, endothelial cells, and macrophages, and that the number of communications in MI was higher than that in donor samples (Figure 9A–D). In the ligand-receptor analysis, multiple signaling pathways were present in MI samples, such as ADIPOQ-ADIPOR2, ANGPTL4 - (ITGA5+ITGB1), NAMPT-INSR, IGF1 -IGF1R, and SEMA3C-PLXND1, among others. Most of these signals were emitted by adipocytes and acted on cardiomyocytes, fibroblasts, endothelial cells (Figure 9E). In contrast, donor samples lacked several ligand-receptor pairs that were specifically active in MI-related pathways, although they retained conserved interactions, such as VEGFA-VEGFR1 and IGF1-IGF1R (Figure 9F). At the level of cellular communication network, these results further validated that disulfidptosis signaling may be involved in the disease process of MI by modulating ligand-receptor interactions between immune cells and cardiomyocytes and endothelial cells.

## 4. Discussion

MI shows high mortality and morbidity, seriously threatening the health of patients [32]. Disulfide bonds form under excessive accumulation of cysteine within cells, leading to the occurrence of disulfidptosis, a newly discovered rapid cell death process [33]. It is imperative to explore the role of disulfidptosis in MI. The present work successfully identified 10 biomarkers closely associated with MI, in particular, *NFIL3* and *MAP3K8* were determined as two signature genes that influenced the proportions of certain immune cells and played crucial roles in the disease mechanism. In vitro experiments further validated the significant role of *MAP3K8* in MI. Additionally, we also predicted 15 drugs closely correlated with these two genes for the treatment of MI.

This study discovered that *NFIL3* and *MAP3K8* were closely linked to disulfidptosis in MI. *NFIL3*, a BZIP transcription factor, is crucial for the development of type 1 innate lymphoid cells (ILC1s) and NK cells [34]. Previous research revealed that *NFIL3* plays a pivotal role in cardiovascular diseases and regulates the pathogenesis of HF [35], functioning as a survival mediator in the heart [36]. *NFIL3* shows a high expression in ischemic myocardial tissue and may be involved in neutrophil-mediated cellular damage [37]. Moreover, the potential of *NFIL3* to serve as a biomarker for MI has also been reported [38]. This present work found that *NFIL3* exhibited a significant negative correlation with multiple T cell-associated immune cell subsets, and that the proportion of T cells was notably reduced in MI samples with high-expressed *NFIL3*. T cell reactivity facilitates myocardial healing by promoting postmitotic organ repair fibrosis [39, 40]. Consistently, the proportion of T cell-related immune genes is significantly lower in the MI group. In accordance with the role of T cells in myocardial reperfusion injury and healing after MI [41], our findings showed that *NFIL3* may affect T cell function in MI. *MAP3K8* is a serine–threonine protein kinase [42] that serves as a major upstream molecule in MAPK (mitogen-activated protein kinase) signal transduction and is capable of activating multiple downstream molecules including *c*-Jun *N*-terminal kinase (JNK), mitogen-activated extracellular signal-regulated kinase (MEK), and extracellular signal-regulated kinase (ERK) to interact with other signaling pathways, such as NF-*κ*B, IL-1, and tumor necrosis factor (TNF) [43, 44]. *MAP3K8* also regulates the number of monocytes, which are pivotal cells in the development of atherosclerosis [45]. In vitro experiments have shown that silencing *MAP3K8* could remarkably inhibit the migratory and invasive capabilities of the cells, indicating a pivotal role of *MAP3K8* in regulating cellular motility and invasion behaviors. In this study, noticeably reduced proportion of T cell-related genes in samples with high-expressed *MAP3K8* also supported a significant negative correlation between *MAP3K8* and T cells, demonstrating that *MAP3K8* can influence the occurrence of MI by negatively regulating the healing response of T cells to the myocardium. Therefore, *NFIL3* and *MAP3K8* were considered as disulfidptosis-related genes that may affect the occurrence of MI by regulating the functions of certain immune cells, such as T cells.

Apart from *NFIL3* and *MAP3K8*, this study had also discovered another eight biomarkers associated with MI, namely, *THBD*, *IRAK3*, *IL1R2*, *THBS1*, *JDP2*, *FCGR2A*, *CCL20*, and *EREG*. *THBD* is a crucial transmembrane glycoprotein constitutively expressed on vascular and lymphatic endothelial cells [46]. Studies found that mutation in *THBD* is a primary cause of thromboembolic diseases [47]. *IRAK3* inhibits toll-like receptor (TLR) signaling to reduce the generation of pro-inflammatory cytokines [48, 49]. Wang et al. performed RNA sequencing and analysis of blood samples from patients with AMI, and found that *IL1R2* and *IRAK3* have potential diagnostic value for AMI [50]. During atherosclerosis and vascular injury, the expression level of *IL1R2* on monocytes/macrophages is downregulated [51]. *THBS1* mediates cell–cell and cell–matrix interactions and is induced in the stroma of many tumors, including atherosclerotic lesions [52], while knocking out *THBS1* can protect the heart from pathogenic stimuli [53]. Moreover, upregulated *JDP2* is closely linked to the progression of HF and the development of atrial arrhythmias [54, 55]. *FCGR2A* is a cell surface receptor on phagocytes, such as neutrophils and macrophages [56]. Study reported a higher expression of *FCGR2A* on platelet surfaces derived from patients with AMI, unstable angina pectoris, and high-risk individuals presenting with two or more atherosclerotic risk factors [57]. *CCL20*, a C─C motif chemokine [53], shows an elevated level in clinical patients with ischemic MI [58, 59]. *EREG* is an autocrine growth factor that activates the MEK/ERK signaling pathway. Silencing *EREG* suppresses angiogenesis and increases ventricular remodeling in AMI rats [60]. The above findings suggested that these 10 biomarkers possessed unique biological functions and mechanisms, and all played crucial roles in the initiation, development, and prognosis of MI and heart-related diseases.

Functional enrichment study on the DEGs revealed that these DEGs were enriched in pathways, such as NF-*κ*B signaling pathway, positive regulation of nitric oxide biosynthetic/metabolic process, IL-17 signaling pathway, chronic inflammatory response, and microglial cell activation. It has been found that after coronary microembolization (CME), TLR4/MyD88/NF-*κ*B signaling pathway may activate the *NLRP3* inflammasome to promote inflammatory cascade and exacerbate myocardial damage [61]. *IRAK3* has also been previously found to be linked to the NF-*κ*B signaling pathway [48, 49]. IL-17 functions critically in immune responses and influences the production of various inflammatory mediators in different cell types, myocardial tissue damage and scarring processes [62]. Furthermore, previous research reported a correlation between nitric oxide signaling and the risk of MI through accelerating thrombosis [63]. MI triggers inflammatory response to clear cellular debris before causing cell death, and excessive inflammation can exacerbate myocardial damage and affect both short- and long-term clinical outcomes [64]. Studies reported increased inflammation in the hypothalamus of patients with MI, accompanied by a significant increase in activated microglia [65]. Microglial depletion alleviates pathological cardiac remodeling after MI by inhibiting neuroimmune responses and sympathetic nerve activity [66]. These DEGs may influence the occurrence of MI through their enrichment in above mentioned MI-related pathways.

However, this study also had several limitations. First, this study mainly relied on a limited sample size of peripheral blood samples from GSE66360 dataset and lacked in situ validation data from myocardial tissues, therefore the current findings may not be able to comprehensively reflect the local molecular changes in the pathogenesis of MI. Our future studies will recruit multicenter clinical samples, and validate the expression and distribution characteristics of the key markers applying tissue-level techniques, such as immunohistochemistry. Second, the GSE66360 dataset is a single data source that lacks CV across multiple cohorts and platforms, which might affect the generalizability and robustness of the findings. In the future, we will combine multicohort data from multiple platforms with machine learning algorithms to construct crossplatform generalizable diagnostic models, and validate them in real-world data. Third, the risk and biological characteristics of MI differed significantly across gender, age groups, and patients with comorbid chronic diseases, but these were not analyzed by stratified modeling in this study. Subsequent studies are encouraged to combine clinical information, construct subgroup-specific diagnostic models, and explore the stability of expression and diagnostic value of the key markers in different populations. Finally, drug intervention experiments remained to be performed to verify the actual biological effects. For this reason, we will carry out drug intervention experiments using an in vitro model to assess the effects of the predicted drugs on the key gene expression and cell function.

## 5. Conclusion

This study successfully identified ten MI-related biomarkers and explored the crucial roles of two particular characteristic genes (*NFIL3* and *MAP3K8*) in the pathological process of MI. We also discovered 15 drugs significantly associated with these the two key genes. Through integrated single-cell transcriptomic profiling and cellular communication network analysis, we further elucidated the expression characteristics of disulfidptosis-related genes in immune cells and their interaction mechanisms with cardiomyocytes and other cells. Our findings provided potential targets and theoretical basis for early diagnosis and immunologic intervention in MI.

## Data Availability

The datasets generated and/or analyzed during the current study are available in the (GSE66360) repository (https://www.ncbi.nlm.nih.gov/geo/query/acc.cgi?acc=GSE66360).

## Nomenclature

MI: Myocardial infarction
CAD: Coronary artery disease
GEO: Gene Expression Omnibus
IRG: Information on immune-related gene
DEG: Differentially expressed gene
WGCNA: Weighted gene coexpression network analysis
TOM: Topological overlap matrix
SVM-RFE: Support vector machines-recursive feature elimination
DMEM/F12: Dulbecco's Modified Eagle Medium/Nutrient Mixture F-12
FBS: Fetal bovine serum
P/S: Penicillin/streptomycin
siRNA: Small intern RNA
OGD: Oxygen–glucose deprivation
PBS: Phosphate-buffered saline
qRT-PCR: Quantitative real-time polymerase chain reaction
CV: Crossvalidated accuracy
LASSO: Least absolute shrinkage and selection operator
ROC: Receiver operating characteristic
GO: Gene Ontology
BP: Biological process
KEGG: Kyoto Encyclopedia of Genes and Genomes
AUC: Area under curve
NK: Natural killer
ILC1: Type 1 innate lymphoid cell
MAPK: Mitogen-activated protein kinase
MEK: Mitogen-activated extracellular signal-regulated kinase
ERK: Extracellular signal-regulated kinase
JNK: *c*-Jun *N*-terminal kinase
NF: Nuclear factor
TNF: Tumor necrosis factor
IL-1: Interleukin-1
TLR: Toll-like receptor
HF: Heart failure
AMI: Acute myocardial infarction
CME: Coronary microembolization.

## Supporting Information

Supporting Information 1 Table S1: Primer sequences used in this study.

Supporting Information 2 Figure S1: Enrichment analysis of highly expressed genes in six cellular subpopulations.
