# Supplementary material for: Identification of Diagnostic Biomarkers for Myocardial Infarction Using Bioinformatics and Disulfidptosis-Targeted Computational Drug Discovery
Source: Mediators Inflamm. 2025 Sep 2;2025:5054377. doi: 10.1155/mi/5054377 (PMC12419921; doi:10.1155/mi/5054377)
Supplement: Supporting Information 1 — Table S1: Primer sequences used in this study. [file 5054377.f1.docx]

Supplementary table 1. Primer sequences used in this study.

| Gene | Forward (5’-3’) | Reverse (5’-3’) |
| --- | --- | --- |
| *MAP3K8* | GCTCTGGCTGGCTACTTCA | CGCTTGGAGGCACATTCAC |
| *NFIL3* | AACTGATGATGGTGTGGTAGGA | TGTGGCAAGGCAGAGGAAT |
| *IL1R2* | CCTGGAAGATGCTGGCTATTAC | TGTGGTGCGTAGTGTCTGAA |
| *CCL20* | TGCTGCTACTCCACCTCTG | GGCTATGTCCAATTCCATTCCA |
| *THBD* | TCCTCTGCGAGTTCCACTTC | GCCGTAGGTGATCGAGACG |
| *IRAK3* | CAGCCAGTCTGAGGTTATGTTT | TTGGGAACCAACTTTCTTCACA |
| *THBS1* | GCCATCCGCACTAACTACATT | TCCGTTGTGATAGCATAGGGG |
| *JDP2* | CCCAGCCCGTGAAAAGTGA | CGGTGTCGGTTCAGCATCA |
| *FCGR2A* | TTTGAGATGAGTAATCCCAGCCA | TCAGGCCCAGTCTCCATTTTA |
| *EREG* | GTGATTCCATCATGTATCCCAGG | GCCATTCATGTCAGAGCTACACT |
| GAPDH | GTCTCCTCTGACTTCAACAGCG | ACCACCCTGTTGCTGTAGCCAA |
