# Supplementary figures and images for: Identification of Diagnostic Biomarkers for Myocardial Infarction Using Bioinformatics and Disulfidptosis-Targeted Computational Drug Discovery
Source: Mediators Inflamm. 2025 Sep 2;2025:5054377. doi: 10.1155/mi/5054377 (PMC12419921; doi:10.1155/mi/5054377)

Pathway Enrichment Analysis

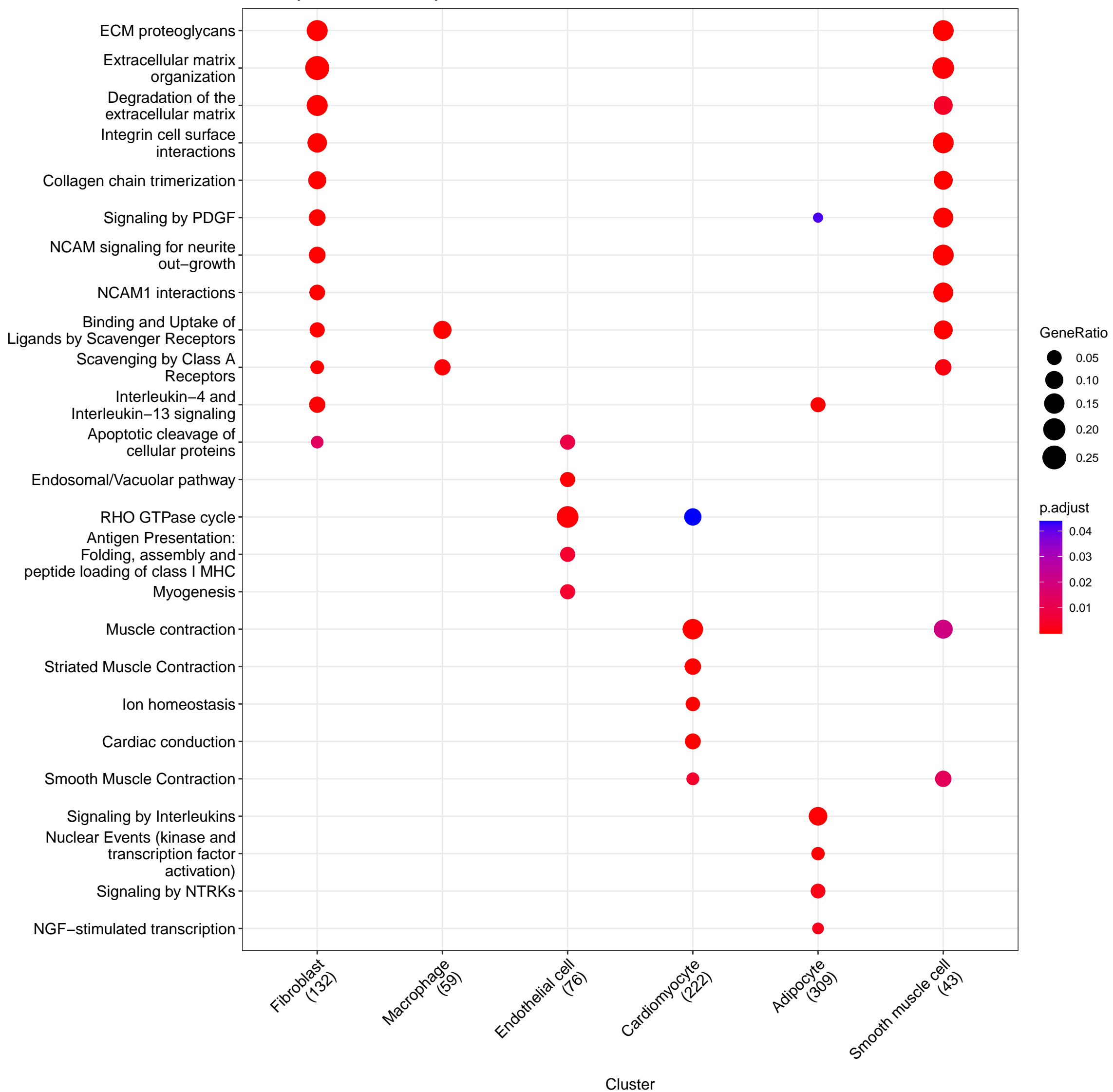

Supplement: Supporting Information 2 — Figure S1: Enrichment analysis of highly expressed genes in six cellular subpopulations. [file 5054377.f2.pdf]
